# Lysosomal exocytosis by macrophages as a druggable mechanism for anti-inflammatory clearance of dead adipocytes in adipose tissue

**DOI:** 10.1038/s41419-025-08334-0

**Published:** 2025-12-23

**Authors:** Raphaela Wehr, Andreas Lindhorst, Lilli Arndt, Martin Krueger, Nora Raulien, Martin Gericke

**Affiliations:** Institute of Anatomy, Leipzig University, Leipzig, Germany

**Keywords:** Diabetes, Obesity, Chronic inflammation, Mechanisms of disease, Lysosomes

## Abstract

The clearance of dead adipocytes in adipose tissue (AT) poses a major challenge due to their large size, which exceeds the phagocytic capacity of macrophages and prevents classical, anti-inflammatory efferocytosis. Instead, adipose tissue macrophages (ATMs) accumulate around dying adipocytes, forming crown-like structures (CLS), and engage in lysosomal exocytosis – the extracellular degradation of adipocytes. In this study, we used an ex vivo explant model of murine epididymal white AT, cultured over seven days to investigate pharmacological strategies that modulate lysosomal exocytosis. We observed a progressive increase in CLS formation, secretion of the lysosomal enzymes ß-Hexosaminidase A (HEXA) and lysosomal acid lipase (LAL), and surface abundance of LAMP1 and LAMP2, confirming ATMs as key mediators of this process. Notably, activation of lysosomal exocytosis with the mTOR inhibitor Rapamycin enhanced adipocyte clearance and significantly reduced inflammatory ATM abundance and TNF-α secretion. Bulk RNA sequencing of ATMs revealed a highly significant impact of Rapamyin on ATM proliferation. In contrast, inhibition of lysosomal exocytosis with PIKfyve inhibitor Apilimod or targeted inhibition of LAL using Lalistat-2 disrupted lysosomal function and promoted a pro-inflammatory ATM phenotype. Our findings highlight lysosomal exocytosis as a critical pathway for the resolution of dead adipocytes and the regulation of inflammation in adipose tissue. Pharmacological enhancement of this process may represent a promising therapeutic approach to attenuate inflammation in AT and its metabolic consequences, including insulin resistance and type 2 diabetes.

## Introduction

Macrophages play a central role in maintaining tissue homeostasis in adipose tissue (AT), where they constitute a significant proportion of total cells. While in lean individuals macrophages make up about 10% of AT cells, the amount increases to approximately 40–50% in obese individuals [1]. Macrophages are vital for the clearance of dead cells and tissue regeneration. This is of utmost importance in AT, with an annual adipocyte turnover rate of ~10% in humans and an indication of an increasing number of dying adipocytes upon high-fat diet (HFD) treatment in mice [2, 3]. Moreover, adipocytes often exceed 100 µm in diameter, much larger than the ~25 µm threshold for macrophage phagocytosis [4]. As a result, effective phagocytosis is insufficient and leads to adipocyte accumulation and locally confined AT inflammation [4] which seems to be the mechanistic link between obesity, inflammation and associated diseases.

Alternative degradation mechanisms besides phagocytosis involve the aggregation of adipose tissue macrophages (ATMs) around dead adipocytes, forming crown-like structures (CLS). Macrophages are traditionally classified into Th1 signal-induced (LPS and IFN-γ) inflammatory M1 and anti-inflammatory M2 types [5, 6]. ATMs in CLS often display a M1-like inflammatory phenotype, with surface markers such as CD11c, CD86, and CD9 [4, 7]. M2 macrophages, prevalent in lean individuals, promote immune regulation and tissue remodeling. They express markers like CD301, CD206 and CD163 [8–12]. Further studies identified a more complex macrophage profile in AT, including metabolically activated macrophages (Mme). Mme are induced by free fatty acids and are characterized by the expression of PLIN2, ABCA1, and CD36, while showing high lysosomal activity [13–15].

Within pro-inflammatory CLS, the proposed degradation pathway of apoptotic adipocytes is extracellular digestion *via* lysosomal exocytosis [16]. Thereby, lysosomes fuse with the macrophage membrane and release enzymes like ß-Hexosaminidase A (HEXA) and lysosomal acid lipase (LAL) into the extracellular space, and the area of the macrophage-adipocyte-interface (lysosomal synapse) becomes acidic, which enables optimal activity of lysosomal hydrolases [16, 17]. Fusion of lysosomes with the ATM membrane is calcium-dependent, with the ion-channel TRPML1 playing a central role. TRPML1 is activated by Phosphatidylinositol-3,5-bisphosphate (PI(3,5)P_2_), which is generated by the lipid kinase PIKfyve [18, 19]. Additionally, lysosomal exocytosis is regulated by transcriptional factors such as TFEB [20, 21].

Hence, our aim was to find a pharmaceutical approach to encourage the anti-inflammatory clearance of adipocytes by macrophages in order to prevent or reduce inflammation caused by accumulating dead adipocytes [1, 22, 23]. Targeting the lysosomal axis, we investigated macrophage activation and lipid degradation. Since LAL is one of the main enzymes secreted during lysosomal exocytosis and of major importance for extracellular lipid degradation, we specifically inhibited it with Lalistat-2 [24, 25].

In our ex vivo model of AT inflammation, we observed increased CLS and a rise in lysosomal exocytosis markers. The surface abundance of the lysosomal-associated membrane proteins was elevated, further supporting the role of lysosomal exocytosis in macrophage activation. Most importantly, our data show that pharmacological intervention of lysosomal exocytosis is sufficient to alter macrophage activation and inflammatory phenotype and could be a target for stimulated resolution of AT inflammation in obese patients.

## Results

**Fig. 1:**
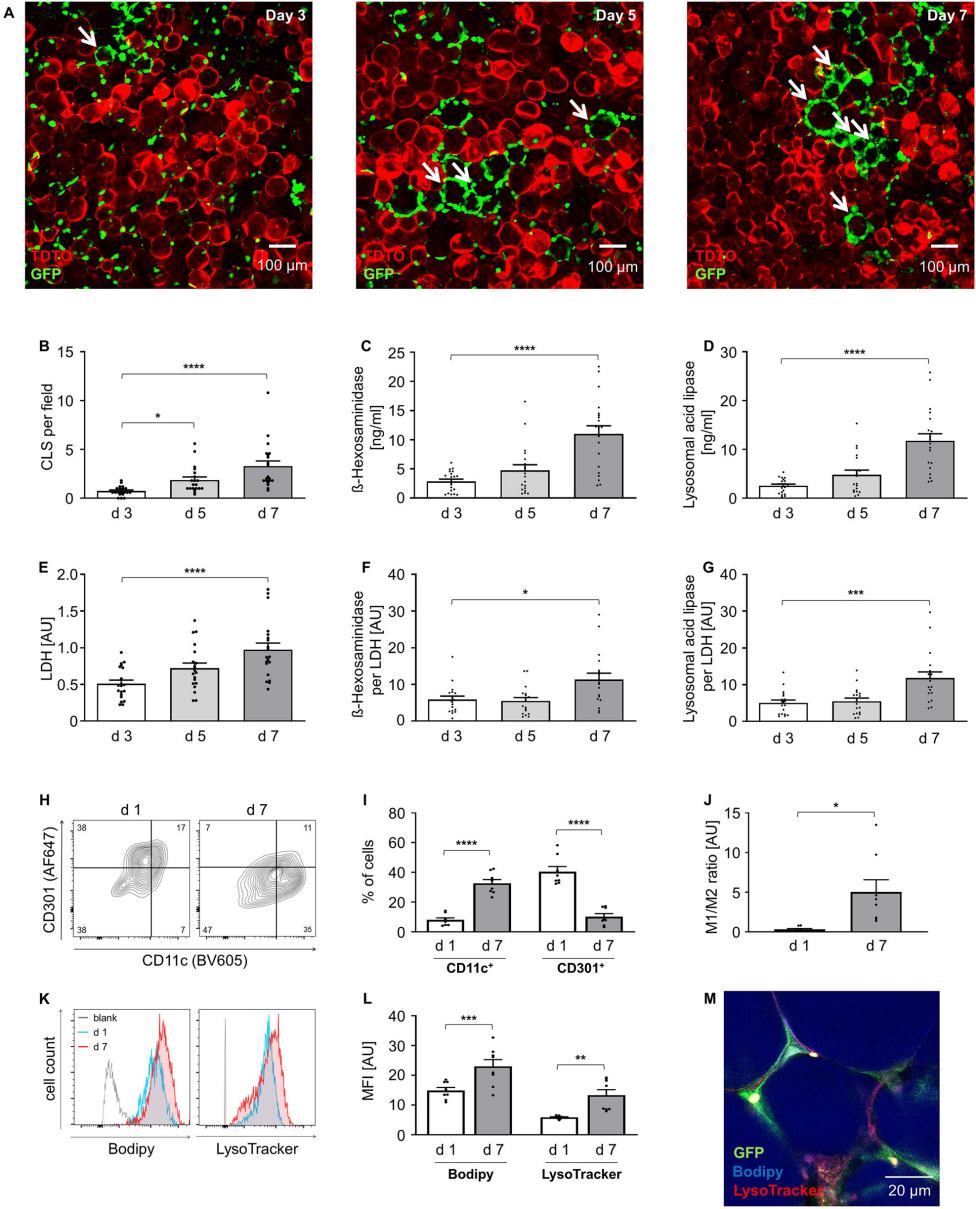
Lysosomal exocytosis in AT-explants. **A** Representative overview confocal microscopy images of AT culture on day 3, day 5 and day 7, ATMs (green), adipocytes (red), arrows indicate CLS. **B** The number of CLS in AT was analyzed per field of view per explant and is shown as mean per well. (*n* = 10) **C–G** ß-Hexosaminidase A, Lysosomal acid lipase and LDH in AT supernatant, determined by ELISA and enzymatic assay per well. (*n* = 10) **H** Representative flow cytometry plots of SVF from day 1 and day 7 of culture, F4/80^+^ / DAPI^−^ cells were classified into CD11c^+^ or CD301^+^ ATMs. **I** Flow cytometry data of CD11c^+^ or CD301^+^ ATMs of day 1 and day 7. (*n* = 8) **J** Ratio of M1 (CD11c^+^) and M2 (CD301^+^) cells. (*n* = 8) **K** Representative flow cytometry histograms of Bodipy or LysoTracker positive ATMs. **L** Flow cytometry data of Bodipy or LysoTracker positive ATMs at day 1 and day 7. (*n* = 8) **M** Representative image of AT explant staining at day 7 of cultivation, ATMs in CLS (green), Bodipy (blue), and LysoTracker (red). All data shown as mean ± SEM, n indicates the number of mice, *p*-value < 0.05 statistically significant (*p < 0.05; **p < 0.01; ***p < 0.001; ****p < 0.0001).

First, we investigated lysosomal exocytosis in an ex vivo model of AT inflammation using murine AT explants cultured for 7 days as proof of concept [26]. CLS formation in AT was analyzed on days 3, 5, and 7 (Fig. 1A). Adipocyte death was traceable due to loss of tdTomato fluorescence beginning at day 3 followed by increased CLS formation at days 5 and 7 (Fig. 1B). This is consistent with previous results showing that CLS develop within 24-48 hours after induced adipocyte death [4]. In parallel with CLS formation, the lysosomal enzymes HEXA and LAL increased (Fig. 1C, D). To rule out excessive cell death we measured Lactate Dehydrogenase (LDH) levels, showing a small increase (Fig. 1E), compared with total LDH release after explant lysis as positive control (Supp. Figure 1A). We calculated the ratio of lysosomal enzymes to extracellular LDH, to exclude necrotic release and still observe significant increased lysosomal enzymes (Fig. 1F, G). In parallel, flow cytometry analysis revealed a shift in macrophage phenotype, with an increasing M1-like (CD11c^+^) and a decreasing M2-like (CD301^+^) population (Fig. 1H–J). This is consistent with previous findings, that inflammatory macrophages, particularly CD11c⁺ cells, accumulate within CLS in obesity models [4, 8, 27, 28]. Additionally, lipid content and intracellular lysosomes increased in ATMs over time (Fig. 1K–M), confirming suitability of our ex vivo model as this mimics the situation in obese AT in vivo.

**Fig. 2:**
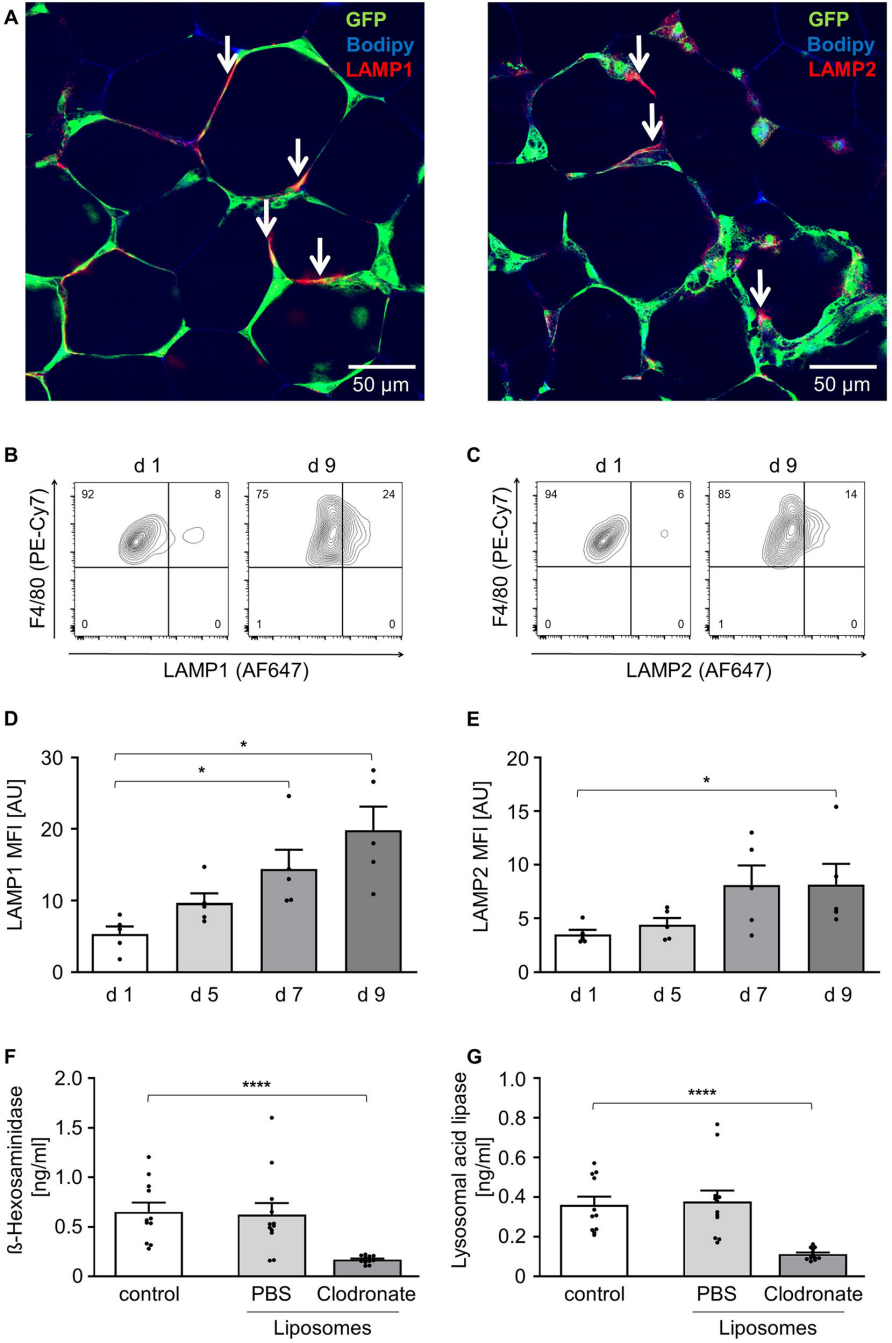
ATMs as source of lysosomal enzymes. **A** Confocal microscopy images of AT explants stained at day 7 of cultivation, ATMs in CLS (green), Bodipy (blue) and lysosomal surface marker LAMP1 or LAMP2 (red, arrows). **B**, **C** Representative flow cytometry plots of F4/80^+^ / LAMP1^+^ or F4/80^+^ / LAMP2^+^ cells at day 1 and day 9 of cultivation. **D**, **E** Flow cytometry data of F4/80^+^ / LAMP1^+^ or F4/80^+^ / LAMP2^+^ cells at day 1, day 5, day 7 and day 9. (*n* = 6) **F**, **G** ß-Hexosaminidase A and Lysosomal acid lipase in AT supernatant at day 7 of cultivation of Clodronate-liposome or PBS-liposome treated AT and untreated AT (control) were determined by ELISA per well. (*n* = 6) All data shown as mean ± SEM, n indicates the number of mice, *p*-value < 0.05 statistically significant (*p < 0.05; **p < 0.01; ***p < 0.001; ****p < 0.0001).

Next, we investigated the role of macrophages as the source of lysosomal enzymes by staining lysosomal associated membrane proteins LAMP1 and LAMP2 (Fig. 2A). Both are primarily involved in lysosomal processes like maintaining lysosomal integrity, pH and catabolism and showed enhanced surface abundance, indicating fusion of lysosomes with the cell membrane (Fig. 2B–E) [29]. To prove that HEXA and LAL are mainly secreted by ATMs, we analyzed the media of AT treated with Clodronate-Liposomes. Clodronate, capsuled in liposomes, efficiently eliminates ATMs in AT explants by ~ 80% [30, 31]. The amount of HEXA and LAL were dramatically reduced in the media of macrophage-depleted AT (Fig. 2F, G), which indicates ATMs as main source of the lysosomal enzymes.

**Fig. 3:**
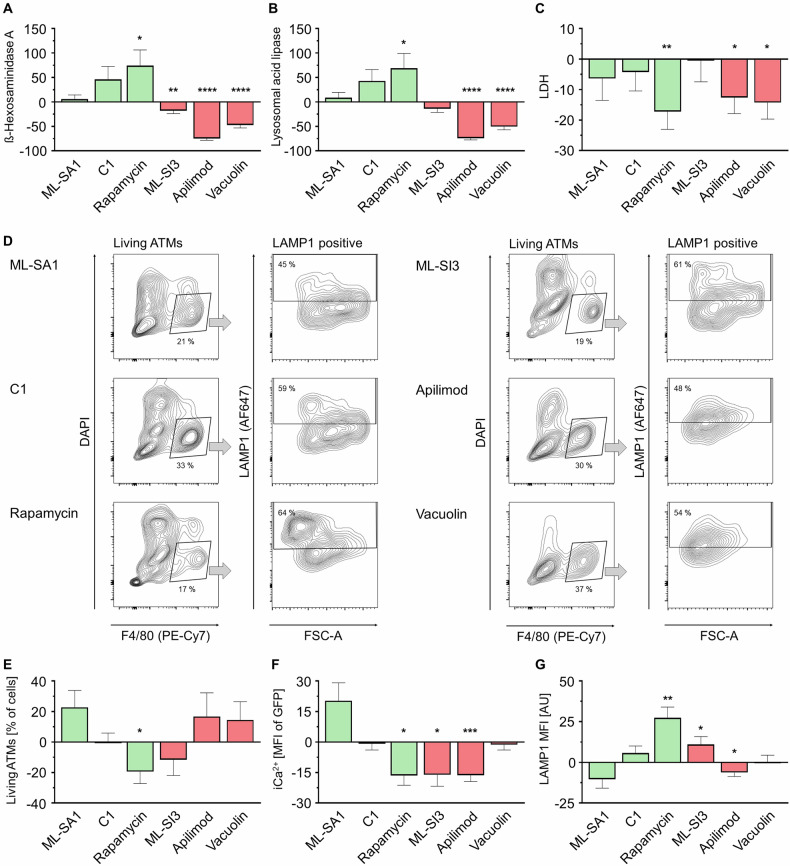
Activation and inhibition of lysosomal exocytosis in AT explants. **A–C** ß-Hexosaminidase A, Lysosomal acid lipase and LDH in treated AT supernatant were determined by ELISA and enzymatic assay per well. (*n* = 8) **D** Representative flow cytometry plots of F4/80^+^ / DAPI^-^ cells (Living ATMs) further analyzed as LAMP1 positive ATMs. **E–G** Flow cytometry data of F4/80^+^ / DAPI^-^ cells (Living ATMs) (**E**), intracellular calcium (iCa^2+^) shown as MFI of GFP in ATMs of CalMac mice (**F**) and lysosomal surface marker LAMP1 MFI (**G**). (*n* = 8) All data shown as mean % of control (DMSO) ± SEM, n indicates the number of mice, *p*-value < 0.05 statistically significant (*p < 0.05; **p < 0.01; ***p < 0.001; ****p < 0.0001).

To modify lysosomal exocytosis in AT explants, we applied different activators and inhibitors of regulatory pathways. Surprisingly, direct TRPML1 treatment with ML-SA1 and ML-SI3 did not show notable effects on ATMs, indicating that this calcium channel plays an inferior role in lysosomal exocytosis or both agents only have a minor mode of action in ATMs (Fig. 3A–G). By blocking endogenous activation of PI(3,5)P_2_ with the PIKfyve inhibitors Apilimod and Vacuolin the release of lysosomal enzymes decreased (Fig. 3A, B). Next, we stimulated TFEB to enhance lysosomal biogenesis in a mTOR dependent (Rapamycin) or independent (C1) manner (Supp. Figure 1B) [21, 32, 33]. Both substances increased lysosomal enzyme secretion, with Rapamycin showing the strongest effect (Fig. 3A, B). Rapamycin treatment reduced the extracellular LDH amount indicating less cell death (Fig. 3C), but it showed the lowest number of ATMs, indicating a reduced ATM activation which results in lower ATM numbers or otherwise an impaired cell proliferation under mTOR inhibition (Fig. 3E). As lysosomal exocytosis is known to be calcium-dependent, we studied intracellular calcium levels (iCa^2+^) using CalMac mice, which express a Ca^2+^-sensitive GFP variant in macrophages. We observed an increase of iCa^2+^ at day 7 of ex vivo culture (Supplementary Fig. 1C). As expected, ML-SA1 increased iCa^2+^ levels, whereas ML-SI3 or Apilimod decreased iCa^2+^. Interestingly, Rapamycin decreased iCa^2+^, confirming that the stimulatory effect on lysosomal exocytosis is more Ca^2+^-independent than previously thought (Fig. 3F). In addition, the LAMP1 surface abundance was affected by exocytosis modulation (Fig. 3D, G). Rapamycin had the strongest effect on increasing surface LAMP1, while the exocytosis inhibitor Apilimod reduced LAMP1, further confirming the results of extracellular enzyme secretion. The effect of Rapamycin on LAMP1 abundance was additionally validated on BMDMs. Surprisingly, Rapamycin increased surface LAMP1 both on Rapamycin-treated BMDMs and on untreated BMDMs co-cultured with Rapamycin-pretreated adipocytes (Supplementay Fig. 1D–F), indicating a more complex crosstalk.

**Fig. 4:**
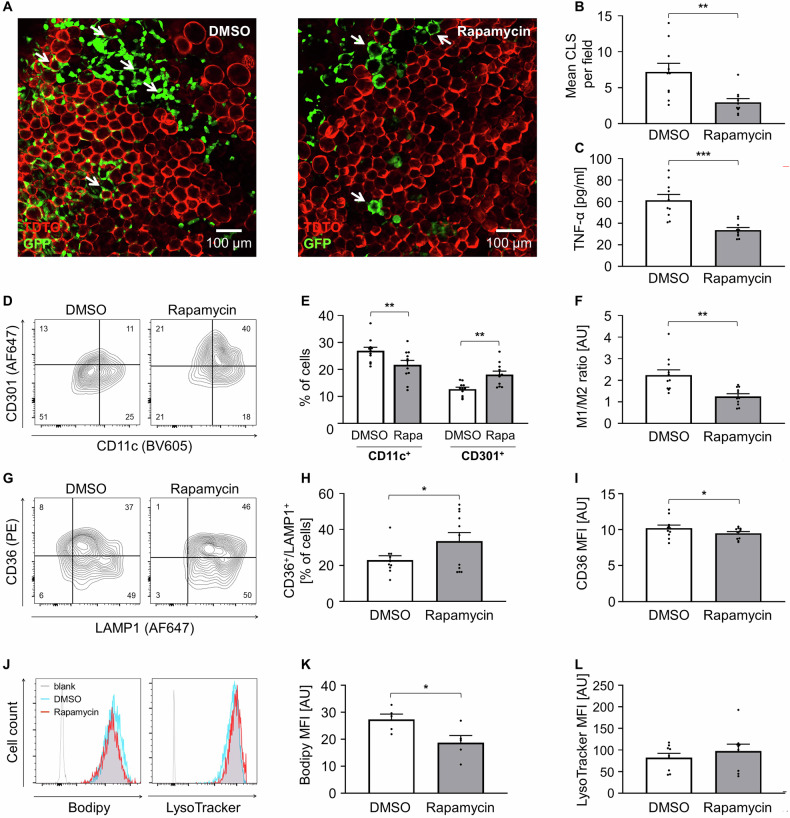
ATM phenotype upon pharmacological influence – mTOR inhibition. **A** Representative confocal microscopy images of DMSO and Rapamycin treated AT culture at day 7, arrows indicate CLS. **B** The number of CLS in DMSO and Rapamycin treated AT was analyzed per field of view per explant and is shown as average per well. (*n* = 5) **C** TNF-α in treated AT supernatant was determined by ELISA. (*n* = 5) **D** Representative flow cytometry plots, F4/80^+^ / DAPI^-^ cells further analyzed as CD11c^+^ or CD301^+^ ATMs. **E**, **F** Flow cytometry data of CD11c^+^ or CD301^+^ ATMs of DMSO- and Rapamycin-treated samples. (*n* = 11) **G** Representative flow cytometry plots of CD36^+^ / LAMP1^+^ ATMs. **H**, **I** Flow cytometry data of CD36^+^ / LAMP1^+^ ATMs of DMSO- and Rapamycin-treated samples. (*n* = 11) **J** Representative flow cytometry histograms of Bodipy or LysoTracker positive ATMs. **K**, **L** Flow cytometry data of Bodipy or LysoTracker positive ATMs of DMSO- and Rapamycin-treated samples. (*n* = 5; *n* = 9) All data shown as mean ± SEM, n indicates the number of mice, *p*-value < 0.05 statistically significant (*p < 0.05; **p < 0.01).

In further experiments, we studied the effects of Rapamycin on ATMs more extensively. Rapamycin significantly reduced CLS formation by ~64% in AT (Fig. 4A, B). Similarly, the secretion of TNF-α, a pro-inflammatory cytokine, was dramatically reduced (Fig. 4C). In line, we found for Rapamycin stimulated tissue a reduced number of CD11c^+^ ATMs and increased CD301^+^ ATMs (Fig. 4D, E). The M1/M2 ratio was therefore lowered (Fig. 4F), indicating a more anti-inflammatory ATM phenotype. We analyzed the concurrent surface abundance of LAMP1 and scavenger receptor CD36, which is a central regulator for lipid metabolism and a marker for Mme (Fig. 4G). CD36^+^/LAMP1^+^ Mme were more present in the Rapamycin samples (Fig. 4H), despite the fact that the CD36 signal was lower for Rapamycin-treated ATMs (Fig. 4I). In line with this finding, a lower Bodipy signal was detected (Fig. 4J, K), showing less lipid content, indicating a more efficient lipid clearing. LysoTracker was used to examine intracellular lysosomes, which tended to be higher in Rapamycin samples (Fig. 4L). Gene expression analysis at day 7 of Rapamycin-stimulated ATMs did not reveal a distinct pattern in regard to macrophage activation. However, KEGG pathway analysis showed significant gene regulation for gene sets involved in TNF signaling or cytokine-cytokine receptor regulation. Most importantly, gene sets for cell cycle regulation were consistently downregulated, explaining the decreased ATM number and the lower TNF-α secretion (Supplementary Fig. 3A, B). Marker genes for M1, M2, and Mme macrophages were inconsistently regulated, whereas there is a slight trend toward upregulation of lysosomal genes (Supplementary Fig. 3C, D).

**Fig. 5:**
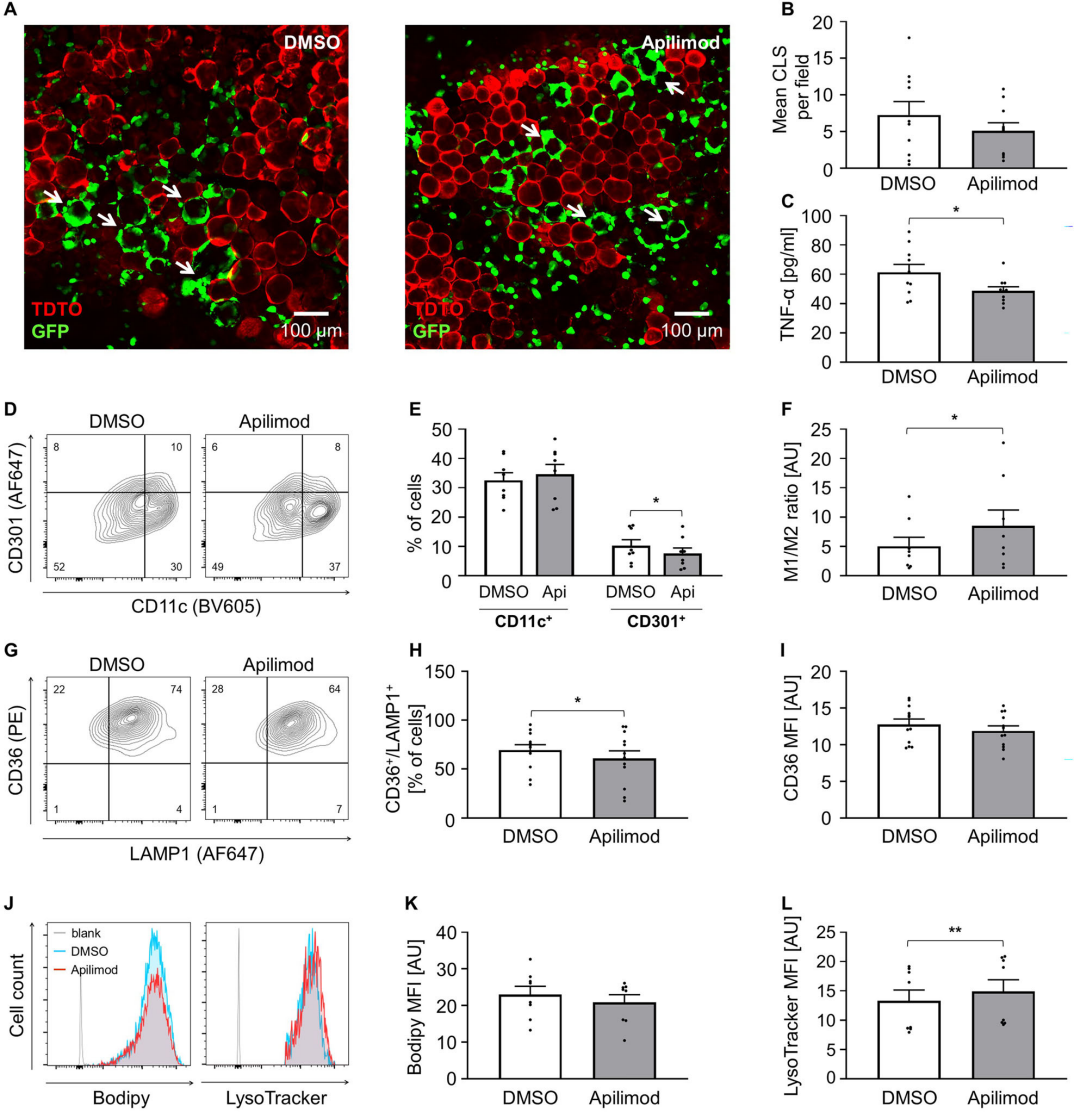
ATM phenotype upon pharmacological treatment – PIKfyve inhibition. **A** Representative confocal microscopy images of DMSO and Apilimod treated AT culture at day 7, arrows indicate CLS. **B** The number of CLS in DMSO and Apilimod treated AT was analyzed per field of view per explant and is shown as average per well. (*n* = 5) **C** TNF-α in treated AT supernatant was determined by ELISA. (*n* = 5) **D** Representative flow cytometry plots, F4/80^+^ / DAPI^-^ cells further analyzed as CD11c^+^ or CD301^+^ ATMs. **E**, **F** Flow cytometry data of CD11c^+^ or CD301^+^ ATMs of DMSO- and Apilimod-treated samples. (*n* = 8) **G** Representative flow cytometry plots of CD36^+^ / LAMP1^+^ ATMs. **H**, **I** Flow cytometry data of CD36^+^ / LAMP1^+^ ATMs of DMSO- and Apilimod-treated samples. (*n* = 12) **J** Representative flow cytometry histograms of Bodipy or LysoTracker positive ATMs. **K**, **L** Flow cytometry data of Bodipy or LysoTracker positive ATMs of DMSO and Apilimod-treated samples. (*n* = 8) All data shown as mean ± SEM, n indicates the number of mice, *p*-value < 0.05 statistically significant (*p < 0.05; **p < 0.01).

Besides activation, we also inhibited lysosomal exocytosis with the PIKfyve inhibitor Apilimod. A change in the CLS number in Apilimod-treated AT was not causal for the reduction of released lysosomal enzymes (Fig. 5A, B). Surprisingly, Apilimod also reduced the inflammatory cytokine TNF-α (Fig. 5C), but did not impact on the abundance of M1 macrophages. Nevertheless, the M1/M2 ratio was more pro-inflammatory with a significant reduction in CD301^+^ cells (Fig. 5D–F). Further, Apilimod caused a significantly reduced number of CD36^+^/LAMP1^+^ Mme, which went in line with reduced lysosomal enzyme release, but with unchanged CD36 expression per cell (Fig. 5G–I). Also, there was no change in the lipid content of the Apilimod-treated ATMs detectable (Fig. 5J, K). Since PIKfyve inhibition is known to prevent fusion of the lysosomes with the ATM membrane and induces vacuole enlargement [34, 35], Apilimod treatment led to a significantly increased LysoTracker signal (Fig. 5L). Likewise, the analysis of the cellular ultrastructure by electron microscopy revealed an increased vesicle size of Apilimod-treated ATMs (Supplementary Fig. 1G, H), confirming feasibility of the intended treatment.

**Fig. 6:**
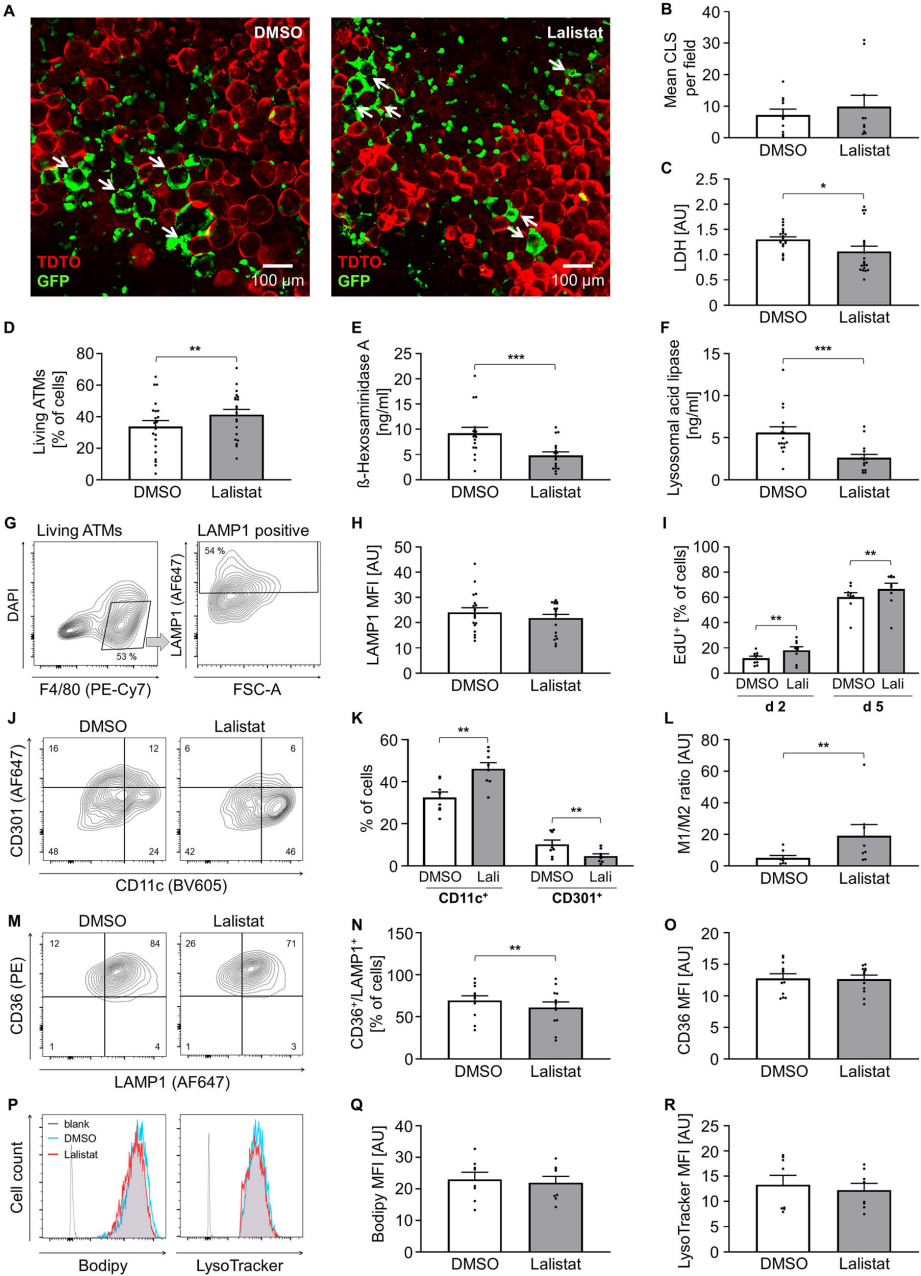
ATM phenotype upon LAL inhibition. **A** Representative confocal microscopy images of DMSO and Lalistat-2 treated AT culture at day 7, arrows indicate CLS. **B** The number of CLS in DMSO and Lalistat-2 treated AT was analyzed per field of view per explant and is shown as average per well. (*n* = 5) **C**, **E**, **F** ß-Hexosaminidase A, Lysosomal acid lipase and LDH in AT supernatant were determined by ELISA and enzymatic assay per well. (*n* = 10) **D** Flow cytometry data of F4/80^+^ / DAPI^-^ cells (Living ATMs). (*n* = 21) **G** Representative flow cytometry plots of F4/80^+^ / DAPI^-^ cells (Living ATMs) further analyzed as LAMP1 positive ATMs. **H** Flow cytometry data: LAMP1 MFI of LAMP1 positive ATMs. (*n* = 19) **I** Proliferation-assay data: EdU^+^ cells of DMSO and Lalistat-2 treated AT on day 2 and day 5. (*n* = 9) **J** Representative flow cytometry plots of CD11c^+^ or CD301^+^ ATMs. **K**, **L** Flow cytometry data of CD11c^+^ or CD301^+^ ATMs. (*n* = 8) **M** Representative flow cytometry plots of CD36^+^ / LAMP1^+^ ATMs. **N**, **O** Flow cytometry data of CD36^+^ / LAMP1^+^ ATMs. (*n* = 12) **P** Representative flow cytometry histograms of Bodipy or LysoTracker positive ATMs. **Q**, **R** Flow cytometry data of Bodipy or LysoTracker positive ATMs. (n = 8) All data shown as mean ± SEM, n indicates the number of mice, *p*-value < 0.05 statistically significant (*p < 0.05; **p < 0.01; ***p < 0.001).

As LAL is one of the main enzymes in lysosomal exocytosis, we used the inhibitor Lalistat-2 to investigate whether the lipid degrading function of LAL is the molecular mediator between lysosomal exocytosis and ATM phenotype. Lalistat-2 had no influence on the CLS number as expected, and only slightly decreased the LDH release, implicating no negative effect on cell survival (Fig. 6A–C). Despite only a minimal reduction in LAMP1 surface abundance the release of the lysosomal enzymes LAL and HEXA was significantly reduced (Fig. 6E, F, H). Lalistat-2 strongly increased ATM number, indicating stimulation of ATM proliferation and activation (Fig. 6D, G). We were able to verify the enhanced proliferation by increased incorporation of EdU-AF350 into new synthesized DNA of proliferating cells (Fig. 6I, Supplementary Fig. 2A). In line, analysis of the ATM phenotype showed more M1-like and less M2-like cells, resulting in a dramatically increased M1/M2 ratio, confirming a pro-inflammatory microenvironment (Fig. 6J–L). However, this pro-inflammatory shift was not accompanied by an increased release of TNF-α (Supp. Fig. 2B). Lower Lalistat-2 concentrations and inhibitors of other lipases did not affect ATM number or phenotype (Supp. Figure 2C–H), indicating a specific effect of Lalistat-2 on LAL and excluding other lipases as mediators in this context. In line with the reduction of lysosomal enzymes, we detected a reduced number of CD36^+^/LAMP1^+^ ATMs for Lalistat-2 treatment (Fig. 6M–O), which points to the presence of fewer Mme, but lipid or lysosomal content and iCa^2+^ in overall ATMs was not affected (Fig. 6P–R, Supp. Figure 2I).

In summary, we here show that with activators and inhibitors of lysosomal exocytosis, we can alter not only the amount of lysosomal enzymes, but also ATM numbers as well as their activation state. Blocking lysosomal function not only reduced adipocyte clearance but also created a pro-inflammatory microenvironment, with elevated ATM numbers and a pronounced shift toward inflammation M1 polarization, most evident under Lalistat-2 treatment. Most importantly, we could show that pharmacological stimulation of lysosomal exocytosis by Rapamycin in AT in the context of an AT inflammation, resulted in a decreased number of ATMs by 20% due to attenuated proliferation, a decreased CLS density by 64%, a more anti-inflammatory ATM phenotype and a reduced intracellular lipid content of ATMs, even with positive effects on overall cell viability.

## Discussion

### Lysosomal exocytosis and macrophage activation in AT inflammation

In obesity, the hypertrophy and hyperplasia of adipocytes, reaching diameters of approximately 100 µm, exceed the macrophages’ anti-inflammatory phagocytic capacity, which is limited to particles up to 25 µm, thereby rendering efficient phagocytic clearance unfeasible [1, 4, 36]. As a result, AT inflammation arises from so-called “frustrated phagocytosis,” which activates macrophages and induces phenotypic changes, like expression of CD11c, and triggers the release of pro-inflammatory cytokines [8, 22, 27]. The inflammatory milieu of AT promotes glucose intolerance and insulin resistance, thereby contributing to the pathogenesis of type 2 diabetes. Elucidating the mechanisms by which macrophages remove dead adipocytes and modulate AT inflammation is therefore essential for identifying novel therapeutic targets. This is particularly pertinent given that current treatments for type 2 diabetes primarily address metabolic dysregulation, while the underlying inflammatory processes remain insufficiently targeted.

In our ex vivo model of AT inflammation, we observed a progressive increase in CLS, indicating ongoing adipocyte death, a hallmark also reported in obesity models in vivo and a key driver of AT inflammation [3, 23]. Enhanced adipocyte death heightens the need for macrophage-mediated clearance and activation, making our system well-suited to identify druggable targets. Elevated levels of the lysosomal exocytosis markers HEXA and LAL, as well as increased abundance of lysosomal surface proteins LAMP1 and LAMP2, suggest that lysosomal exocytosis is the primary mechanism by which dead adipocytes are cleared in AT. Based on these findings, we aimed to further investigate the process, assess pharmacological strategies to promote anti-inflammatory degradation of adipocytes, and gain a deeper understanding of the role of macrophages.

To this end, we tested activators and inhibitors targeting the regulatory axis of lysosomal function. First, we examined ML-SA1, a TRPML1 activator, and C1, a TFEB activator, both of which have been proposed to enhance lysosomal exocytosis [18, 19, 37, 38]. Although ML-SA1 did increase intracellular calcium levels, it did not affect lysosomal exocytosis. Other calcium channels, such as the Two-pore channels, e.g. TPC2 and the calcium sensor Synaptotagmin VII, are likely to play a pivotal role in regulation [39, 40]. Nevertheless, ML-SA1 remains a promising candidate for inducing autophagy and lysosomal biogenesis in other cells and contexts [41–43].

However, we cannot exclude that the limited efficacy of some pharmaceuticals in our model may be due to poor diffusion into the tissue, possibly related to their molecular size or structural properties. Additionally, degradation of the compounds through unknown mechanisms cannot be excluded. Due to methodological constraints, we were unable to assess pharmacokinetics or pharmacodynamics in detail; however, based on our data, a substantially greater effect in vivo appears unlikely.

### Rapamycin promotes lysosomal exocytosis and an altered ATM polarization

Among the compounds tested, the mTOR inhibitor Rapamycin emerged as an activator of lysosomal exocytosis. Rapamycin is an immunosuppressant, used post-transplantation, and has anti-tumor properties [44, 45]. Rapamycin is supposed to activate TFEB *via* mTOR-dependent pathways and has also been shown to bind TRPML1 and TPC2. However, the exact mechanism through which Rapamycin promotes lysosomal processes remains unclear. In our AT explant model, Rapamycin increased lysosomal exocytosis and importantly altered ATM phenotype. This observation supports prior evidence that Rapamycin induces a M2-like macrophage phenotype across other biological contexts, with polarization mediated *via* the PI3K-AKT-mTOR axis [46, 47]. The anti-inflammatory microenvironment was further characterized by lower TNF-α levels, a reduced ATM number and a decreased CLS density along with improved cell viability (evidenced by lower LDH release). Gene set analyses indicated a mixed picture of up- and downregulated inflammatory genes. However, a decrease in ATMs and an attenuated inflammation was also observed by Jian et al. in *Mac-Raptor* ^KO^ mice [48]. Noteworthy, the lower number of ATMs could be due to reduced cell proliferation by Rapamycin, which we also confirmed by our gene expression analysis after bulk RNA sequencing. This effect is mediated through complex formation with FKBP12, which suppresses cellular growth [49, 50]. In addition to the elevated LAMP1 signal on ATMs, we detected a modest increase in LAMP1 on the surface of Rapamycin-stimulated BMDMs. The underlying mechanisms remain unclear. Co-cultures where only isolated adipocytes were pretreated with Rapamycin also led to an increase of LAMP1 on BMDM surface. Previous studies have shown that Rapamycin promotes lipid release from adipocytes [51], and we likewise observed higher levels of free fat in Rapamycin treated samples (unpublished observation). Thus, we hypothesize that increased abundance of free lipids in co-cultured BMDMs contributes to the elevated LAMP1 signal due to metabolic activation of BMDMs after lipid stimulation as described earlier [14]. Additionally, the decreased Bodipy signal indicates a reduction in ATMs’ intracellular lipid content. This is consistent with increased lysosomal lipid degradation – one of the intended outcomes of lysosomal activation. We cannot fully exclude that the observed reduction in lipid content is due to decreased lipid uptake, potentially reflecting reduced CD36 surface abundance. CD36 facilitates fatty acid uptake and lipid metabolism and is a hallmark of lipid-laden, lysosomal active macrophages (MMe) [52]. Because Rapamycin promotes lysosomal activity, we anticipated an expansion of MMe and indeed detected an increase in CD36⁺/LAMP1⁺ ATMs following treatment. This suggests that Rapamycin not only enhances lysosomal exocytosis but also shifts ATM composition toward MMe, thereby linking improved lipid handling with an anti-inflammatory ATM phenotype.

Although the broad systemic effects and known side effects of mTOR inhibition are challenges for its application in obesity-associated AT inflammation and insulin resistance, Rapamycin still offers valuable mechanistic insights. These findings highlight the potential for developing selective modulators of lysosomal function within AT, warranting further investigation.

### PIKfyve inhibition disrupts lysosomal exocytosis and increases ATM inflammation

We were also interested in inhibiting lysosomal exocytosis to gain further insight into its relevance for AT inflammation. Since TRPML1 inhibition only had a minor effect, we focused on alternative pathways, including inhibition of PIKfyve, as described above [53]. In diabetes context, studies found that PIKfyve is essential for glucose metabolism and insulin sensitivity [54, 55]. PIKfyve inhibition by Apilimod blocks the fusion of lysosomes with the macrophage membrane, thereby preventing extracellular release of lysosomal enzymes. Reduction of PIKfyve impairs the activity of lysosomal ion channels, including TRPML1, TPCs, and V-ATPase [19, 56, 57]. As previously reported, PIKfyve inhibition induced cellular vacuolation and expansion of endo- and lysosomal compartments, accompanied by reduced Ca²⁺ levels in ATMs [58, 59]. Correspondingly, we observed an accumulation of enlarged lysosomes. Apilimod is known to suppress IL-12 and IL-23 expression and has thus been evaluated in clinical trials for the treatment of inflammatory diseases [60–62]. However, our data did not reveal a clear impact on ATM activation state, as Apilimod slightly reduced both M2 and Mme, as well as TNF-α in the supernatant. As shown before, TNF-α is preferentially secreted by M1 and Mme [14]. Hence, a reduction of Mme as the most abundant ATM subset ( > 60%), may be sufficient to attenuate TNF-α secretion. This result underscores the complexity of macrophage polarization in AT. Although lysosomal exocytosis was reduced, likely limiting extracellular lipid availability, intracellular lipid content and CD36 abundance remained unchanged, indicating a potential delay rather than a complete impairment in adipocyte clearance. Moreover, the clearance of dead adipocytes is likely a gradual process, potentially taking several months [36, 63]. Detecting changes in CLS density or ATMs lipid burden may require longer-term and high-resolution analyses. Notably, ATM immune phenotypes may respond more rapidly to shifts in lipid composition, highlighting the value of integrating cytokine profiling and detailed lipidomics in future studies.

### Disruption of lysosomal exocytosis *via* LAL blockade induces pro-inflammatory macrophage activation

LAL is a key lysosomal enzyme and the only known intracellular lipase that functions at acidic pH. It can be selectively inhibited by Lalistat-2, a compound used to detect LAL deficiency [64]. Interestingly, in addition to reducing LAL activity, Lalistat-2 treatment decreased secretion of HEXA, suggesting a broader impact on lysosomal enzyme trafficking or release. Functionally, LAL inhibition promotes a pro-inflammatory ATM phenotype and strongly enhanced ATM proliferation. While the cellular mechanisms underlying cell proliferation remain unclear, previous studies have linked ATM proliferation with more severe AT inflammation, mediated in part by IL-6 signaling in obese mice [65, 66]. In general, inefficient efferocytosis is associated with increased proinflammatory cytokine production and polarization towards a M1-phenotype [67]. Consistent with these findings, LAL deficiency and LAL knockout models have been shown to drive systemic inflammation across various organs, with macrophages playing a central role [68]. However, in our model, ATM lipid content remained unchanged. This may be due to the short experimental timeframe, as lipid accumulation is typically observed in long-term Lalistat-2 treatments or genetic models of LAL deficiency [69]. Alternatively, the apparent discrepancy could be explained by the lipid-rich environment of our AT model and potential upregulation of compensatory lipases following LAL inhibition.

In summary, our results demonstrate that the number of CLS increases over time in AT explants, alongside a rise in lysosomal exocytosis markers. This supports the use of AT explants as a relevant model to study druggable mechanisms involved in AT inflammation, particularly lysosomal exocytosis. The increased abundance of LAMP1, LAMP2, and LysoTracker indicates that ATMs are the main source of lysosomal enzymes and lysosomal exocytosis is the predominant mechanism for the clearance of dead adipocytes. Importantly, enhancing lysosomal exocytosis with mTOR-inhibitor Rapamycin improved adipocyte clearance and reduced CLS, highlighting the therapeutic potential of this pathway. However, direct effects of Rapamcyin on macrophages and adipocytes were also shown and need to be considered (Suppl. Figure 1D). Conversely, inhibition of lysosomal exocytosis using Apilimod, or LAL inhibition with Lalistat-2, impaired lysosomal function. Although the effects of Apilimod and Lalistat-2 on ATM activation were less pronounced, they indicated a role for lysosomal lipid degradation in ATM proliferation. This is because increased ATM numbers after Lalistat-2 stimulation and decreased ATM numbers after Rapamycin stimulation corresponded to stimulated or attenuated ATM activation, respectively.

Together, these findings demonstrate that lysosomal exocytosis not only facilitates the degradation of dead adipocytes but also modulates macrophage activation and polarization. Targeting this pathway may offer a novel strategy to counteract chronic inflammation in AT and associated metabolic disorders.

## Material and methods

### Mice

All mice were housed in a temperature-controlled (22 ± 2 °C), pathogen-free facility in sibling groups of 3–5 mice on a 12 h light/dark cycle. Mice were fed a standard normal chow diet (9% kcal fat, Sniff GmbH; Germany) with free access to water. To visualize macrophages in vivo, macrophage reporter mouse lines were used: *Csf1r-*eGFP reporter mice (“MacGreen”) [70], LyzMCre-Cre x tdTomato (“LysTomato”) [71, 72] or LyzMCre-Cre x GCaMP3, with a Ca^2+^-sensitive GFP variant, (“CalMac”) [71, 73]. For representative microscopy-images *Csf1r*-eGFP-*Adipoq*-ERT2-Cre-*CAG-tdTomato* mice (“MacFat”) [70, 74] were used to visualize adipocytes due to tdTomato expression. All mice were on a C57BL/6 J background. Animal experiments followed the ‘Principles of laboratory animal care’ and were approved by the authorities of the state of Saxony (T11-21-MEZ; Landesdirektion Leipzig, Germany).

### Adipose tissue explant culture

For CLS induction, we used an ex-vivo tissue culture model [26]. After sacrificing the mice, the rostral eWAT was dissected under sterile conditions and cut into small pieces (explants) of ≈ 1 mm^3^ (10 mg per explant) in PBS. Five AT explants per well were transferred to six-well plates prepared with 1 ml cell culture medium: RPMI 1640 (Sigma Aldrich, Steinheim, Germany) with 10% fetal bovine serum (FBS, Gibco, Life Technologies, Carlsbad, CA, USA) and 1% antibiotics (100 U/ml penicillin and streptomycin, Gibco). Explants were overcast with cell culture inserts and cultured at 37 °C with 5% CO_2_ and 21% O_2_ for seven days.

### Culture of bone marrow derived macrophages (BMDMs)

Bone marrow of adult MacGreen mice was flushed with cold PBS from femurs and tibiae. The cell solution was filtered through a 70 µm cell strainer (Sarstedt) and centrifugated (300 *g*, 5 min, 4 °C). The cell pellet was resuspended with media and cell suspension was added in untreated culture dishes and cultured at 37 °C with 5% CO_2_ and 21% O_2_ for seven days. BMDMs were differentiated in RPMI1640 (Thermo Fisher Scientific) supplemented with 1 mM GlutaMax, 10% FBS, 1% Penicillin/Streptomycin (all from Gibco), and 20 ng/ml M-CSF (PeproTech, Hamburg, Germany). At day 3 and 5 fresh M-CSF and culture media was added. BMDMs were harvested at day 7 with ice-cold 1 mM EDTA/PBS solution.

### Adipocyte isolation and culture

Epididymal fat pads were excised from adult male mice as previously described and transferred into sterile PBS. Approximately 100 mg of AT was transferred into 2 ml tubes containing 1.5 ml of digestion buffer (see collagenase digestion as described below) and finely minced with a scissor. Tissue digestion was carried out at 37 °C for 15 min with constant shaking at 1400 rpm. Digestion was stopped by adding 150 µl FBS, the suspension was filtered through a 300 µm mesh. Adipocytes were washed with isolation buffer (PBS + 10% FBS) and centrifuged (100 *g*, 3 minutes, 21 °C). The infranatant was carefully removed using a long needle attached to a syringe. Washing was repeated 2–3 times with decreasing buffer volumes.

For culture, adipocytes were gently resuspended and pipetted into the center of a six-well plate using a cut pipette. A cell culture insert (Sarstedt) was placed on top of the suspension, and culture medium (DMEM, supplemented with 10% FBS and 1% penicillin-streptomycin) was added below and above the insert.

### Co-culture of BMDMs with adipocytes

BMDMs were differentiated as described above. After harvesting cell number was determined and cells were seeded in a 6-well plate (1.5 × 10^6^ cells/well). BMDMs adhered for 30 min, then the medium (RPMI 1640 with 1 mM GlutaMax, and 1% Penicillin/Streptomycin) was removed and adipocyte cell suspension was pipetted into the center of each BMDM covered well and over casted with an insert (Sarstedt). 1 ml culture medium (DMEM, supplemented with 10% FBS and 1% penicillin-streptomycin) was carefully pipetted below and above the insert. The cells were co-cultivated for 24 h (37 °C, 5% CO_2_, 21% O_2_).

### Pharmacological treatment of AT explants, BMDMs and adipocytes

All chemicals were dissolved in DMSO and further diluted with cell culture media. On day one of AT explant culture, 100 µl treatment solution was added in each well. Chemical concentrations were determined *via* concentration series. As activators ML-SA1 10 µM (Sigma Aldrich), TFEB activator 1 (C1) 2 µM (TargetMol, Wellesley, MA, USA), Rapamycin 0.5 µM (TargetMol) and Resveratrol (RSV) 5 µM (TargetMol) were used. ML-SI3 10 µM (MedChem Express, Monmouth Junction, NJ, USA), Apilimod 0.5 µM (Sigma Aldrich) and Vacuolin-1 5 µM (Cayman Chemical, Ann Arbor, MI, USA) were used as inhibitors. Lalistat-2 10 µM (Cayman Chemical) was used to inhibit LAL, with concentrations of 1 µM and 0.1 µM also tested. Atglistatin 50 µM (MedChem Express), an adipose triglyceride lipase (AGTL) inhibitor and Hi-760079 25 µM (MedChem Express), a hormone-sensitive lipase (HSL) inhibitor, were also tested.

BMDMs were treated with Rapamycin 0.5 µM and corresponding DMSO concentration (0.01%) on day 5 of cell culture. The stock solution was added directly to the fresh cell culture medium which was added as planned on day 5.

Adipocytes were pretreated with Rapamycin 0.5 µM and corresponding DMSO concentration (0.01%) for 8 h. Then the cells were washed with buffer (PBS with 10% FBS). Adipocytes were added to adherent untreated BMDMs and both were co-cultivated for 24 h.

To ensure that ATMs were not impacted by the vehicle solution DMSO, we used identical DMSO concentration for all samples and investigated the influence of DMSO on ATMs with flow cytometry. (Supp. Figure 2J) We agreed on 0.5% DMSO as the maximum compatible concentration in the AT explant culture.

### Clodronate liposome treatment

Macrophages in AT explants were depleted as described previously by our group [30, 75]. Clodronate-liposome treatment eliminates more than 80% of the ATMs, as determined by flow cytometry [31]. PBS, which was encapsulated in liposomes, was used as a liposome-control. The PBS-liposomes did not reduce the amount of living macrophages in the tissue. The supernatant of Clodronate- or PBS-liposomes treated AT and untreated AT (control) was analyzed with ELISA for lysosomal enzymes HEXA and LAL.

### Confocal microscopy

To monitor AT and CLS formation, living AT was analyzed on days 3, 5, and 7 of organ culture using confocal microscopes FV1000 (Olympus Deutschland GmbH, Hamburg, Germany) and Leica SPE microscope (Leica, Wetzlar, Germany), both equipped with internal lasers. A 10x objective was used for overviews and a 40x objective for detailed analyses.

### Electron microscopy

Cultivated and treated AT was fixed with 4% paraformaldehyde (Serva, Heidelberg, Germany) and 1.5% glutaraldehyde (Serva), then stained with 0.5% osmium tetroxide (EMS, Hatfield, PA, USA) in PBS for 30 min. After PBS rinsing, the tissue was dehydrated in 30%, 50%, and 70% ethanol. Afterwards, the explants were treated with 1% uranyl acetate (Merck, Darmstadt, Germany) in 70% ethanol for 1 h, and further dehydrated in 80%, 90%, 96%, 100% ethanol, and propylene oxide (Sigma Aldrich). Explants were embedded in Durcupan (Sigma Aldrich), trimmed, and cut into 55 nm slices using an ultra-microtome (Leica Microsystems). Slices were placed on Formvar-coated grids and stained with lead citrate. A Zeiss SIGMA electron microscope (Zeiss NTS, Oberkochen, Germany) was used for imaging, and vesicle size was analyzed with ImageJ.

### Whole mount staining of AT

AT was stained on day 7 of cultivation. After PBS washing, explants were fixed in zinc formalin (Polysciences, Hirschberg, Germany), washed with PBS, blocked with staining buffer (3% bovine serum albumin (BSA) in PBS) for 1 h, and stained with antibodies in staining buffer (4 °C, overnight). Neutral lipids in adipocytes were stained with 1 µg/ml BODIPY 558/568 C12 (1:10,000, Life Technologies). Lysosomes were labeled with LysoTracker™ Deep Red (1:2000, Thermo Fisher Scientific, Darmstadt, Germany). Lysosomal surface markers on ATMs were visualized using Alexa Fluor 647-conjugated LAMP1 (1:100, Biolegend, San Diego, CA, USA) and LAMP2 (1:100, Biolegend) antibodies.

### ELISAs and assays

The amount of HEXA and LAL was measured in AT supernatant, maintaining a constant AT-to-media ratio (50 mg AT per 1 ml media). Enzyme levels were determined using ELISA-kits (both abbexa, Cambridge, UK [abx254518 and abx573100]). LDH was determined with CyQUANT LDH Cytotoxicity Assay Kit by Invitrogen (Waltham, MA, US). TNF-α levels in AT supernatant was determined using BD OptEIA™ Mouse TNF ELISA Kit. All samples were analyzed in duplicates.

Total LDH was assessed after forced AT lysis using 1 mg/ml collagenase type 2 ( ~ 315 U/ml; Worthington, USA) in 1 ml RPMI 1640 with 1% Triton-X 100 (Carl Roth, Germany), shaking at 1400 rpm at 37 °C for 20 min. The reaction was stopped with 100 µl FBS, and the clear phase below the fat layer was snap-frozen.

Macrophage-proliferation was analyzed with the Click-iT™ Plus EdU Alexa Fluor™ 350 Assay Kit by Invitrogen. ATMs were labeled with 5 µM Click-iT EdU on day 1, adding 100 µl of EdU stock solution to the culture medium. At day 7, AT was digested, cells were fixed, and EdU was detected. Measurements were performed on the BD LSRFortessa™ Cell Analyzer (BD Bioscience, Heidelberg, Germany) as described below.

### Collagenase digestion

AT explants were digested by 1 mg/ml collagenase type 2 (Worthington) in digestion buffer (13 mM HEPES; 0.8 mM ZnCl_2_; 3% BSA in HBSS) for 15–20 min at 37 °C, shaking at 1400 rpm, to extract the stromal vascular fraction (SVF). The process was stopped with 10% FBS. The suspension was filtered through a 70 µm mesh. The cell pellet was washed twice with staining buffer after centrifugation (300 *g*, 10 min, 4 °C, no break).

### Flow cytometry analysis

After washing, the SVF was resuspended in staining buffer and Fc-receptors were blocked with anti-CD16/32 (1:100, eBioscience, Frankfurt, Germany) (10 min, dark, on ice) followed by washing. Cells were stained with antibodies (Supplemental table 1) for 20 min on ice and washed, centrifuged (300 *g*, 5 min, 4 °C, slow deceleration), resuspended in staining buffer and kept on ice until flow cytometry. DAPI (0.2 µg/ml, Thermo Fisher) or 7-AAD (1:100, BD Pharmingen, BD Bioscience) were used for excluding dead cells.

Measurements were performed on the BD LSRFortessa™ (BD Bioscience) with FACS Diva software 9.01 at Leipzig University’s Core Unit ‘Fluoreszenz Technologien’.

Single cells in SVF were gated for DAPI negative/ 7-AAD negative cells and F4/80^+^ cells were defined as ‘Living ATMs’ (see gating strategy, Isotypes, blanks and FMOs: Supplementary Figs. 4 and 5). The ‘% of cells’ refers to the proportion of living ATMs in the SVF SSC-A/FSC-A-fraction. For CD301^+^ or CD11c^+^ or CD36^+^ / LAMP1^+^ ATMs, ‘% of cells’ refers to the proportion of the respective cells in the Living ATMs. With the MFI data shown above, the real values are x10^3^ higher. Quantification used FlowJo software 10.10. (BD Bioscience).

### Western blot analysis

Following AT digestion, the SVF was washed twice before lysis and fractionation into nuclear and cytosolic proteins. The cell pellet was incubated for 15 min on ice in extraction buffer (20 mM HEPES, 10 mM KCl, 2 mM MgCl_2_, 1 mM EDTA, 1 mM EGTA, 1 mM DTT, 1% protease inhibitor mixture [1:100, Cell Signaling Technology Inc., Danvers, MA, USA]) and then lysed by passing through a 27-gauge needle 10 times. After 20 min incubation on ice and centrifugation (720 *g*, 5 min, 4 °C) the supernatant contains the cytosolic fraction. The cell pellet was washed and passed through a 25-gauge needle 10 times, after centrifugation resuspended in wash buffer (TBS) with 0.1% SDS and sonicated to shear genomic DNA and homogenize (nuclear fraction).

Protein concentration was determined using Bradford Assay protocol with the Biorad Protein Assay Dye Reagent Concentrate (Biorad, Hercules, CA, USA) and BSA as standard (2 mg /ml series; Sigma Aldrich). SDS page was performed using a 4–15% gradient gel (Biorad). Proteins were transferred *via* semi-dry blotting with the Trans-Blot® Turbo™ Transfer System (Biorad) on the nitrocellulose membrane. Membranes were blocked in 5% fat-skimmed milk in TBS (5 mM Tris, 15 mM NaCl, 3% Tween 20 in Aqua dest.). The primary antibody (1:5000, rabbit anti-TFEB; Bethyl-Laboratories, Montgomery, TX, USA) was incubated overnight at 4 °C, and then detected with peroxidase-conjugated anti-rabbit IgG-HRP secondary antibody (1:10.000, PI-1000, Vector Laboratories, Newark, CA, USA). The peroxidase reaction was visualized with ECL kit (Thermo Fisher Scientific). Loading controls were performed with anti-Lammin B1 antibody (1:1000, rabbit mAb; Abcam, Cambridge, UK, nuclear marker) and anti-GADPH antibody (1:1000, mouse mAb; Fitzgerald/ Biosynth Ltd, Compton, UK, cytosolic marker).

### Bulk RNA sequencing

Treated AT explants were digested using collagenase as described above. SVF was resuspended in staining buffer and the Fc-receptors blocked by anti CD16/32 (1:100, eBioscience, Frankfurt, Germany) for 10 min on ice. After centrifugation (300 *g*, 5 min, 4 °C, slow deceleration) cells were stained with labeled F4/80 antibody (BioLegend, San Diego, USA) on ice for 20 min, centrifugated (300 *g*, 5 min, 4 °C, slow deceleration), resuspended in staining buffer. Living, F4/80^+^ cells were sorted ( ~ 50,000 cells per sample) using a BD FACSAria SORP (Becton Dickinson, Franklin Lakes, USA) and frozen at −80 °C in TRIzol (Thermo Fischer Scientific). RNA isolation and purification was performed using RNeasy Micro Kit by Quiagen (Venlo, The Netherlands).

RNA sequencing and analysis was performed at Leipzig University’s Core Unit ‘DNA Technologien’. RNA extraction and library preparation was performed with a sequencing depth of 10 million reads/sample. Results were then analyzed using the DESeq2 package on Galaxy2 [76]. Differentially expressed genes (DEG) were considered as significant with a p-adj. value < 0.01 and a log2(fold change) > 0.5 or < -0.5. Gene set enrichment analysis was performed with datasets from the DAVID Database. Shown are a KEGG pathway analysis of significant regulated genes and heatmaps, with z-scores of DEGs in Rapamycin samples compared to DMSO control. For each condition, AT from 8 animals was pooled in groups of two, so that 4 samples were analyzed for each treatment. RNASeq data are available at GEO database (GSE307090).

### Statistical analysis

Data are shown as mean ± SEM or % of control ± SEM (Fig. 3). Data sets were tested for statistical outliers with the ROUT test (1%) and normality using the Shapiro-Wilk test (significance level 0.05) using GraphPad Prism 10.2 (GraphPad Software Inc., La Jolla, USA). Statistical significance analysis was performed with paired Students-T or Wilcoxon tests. Multiple conditions were compared using one-way ANOVA followed by Dunnett’s post hoc test. *P*-values < 0.05 were considered significant. In all figures, *n* refers to the number of biological replicates (i.e., individual mice). The sample size depended on the type of experiment, but was always at least *n* = 5, with the exception of gene expression analysis (*n* = 4).

### AI statement

AI-used Large language models (ChatGPT) were used to shorten the text by a few words. ChatGTP and Deepl were used for wording suggestions and translations. However, the text was always written by the first author.

## Supplementary information

Supplemental Figure 1

Supplemental Figure 2

Supplemental Figure 3

Supplemental Figure 4

Supplemental Figure 5

Supplemental Figure Legends

Table 1 Supplemental information

TFEB WesternBlot

## Data Availability

Data sets generated during the study are available from the corresponding author on request. RNASeq data sets are available at GEO database (GEO accession: GSE307090).
