# Supplementary figures and images for: Lysosomal exocytosis by macrophages as a druggable mechanism for anti-inflammatory clearance of dead adipocytes in adipose tissue
Source: Cell Death Dis. 2025 Dec 23;17(1):124. doi: 10.1038/s41419-025-08334-0 (PMC12848297; doi:10.1038/s41419-025-08334-0)

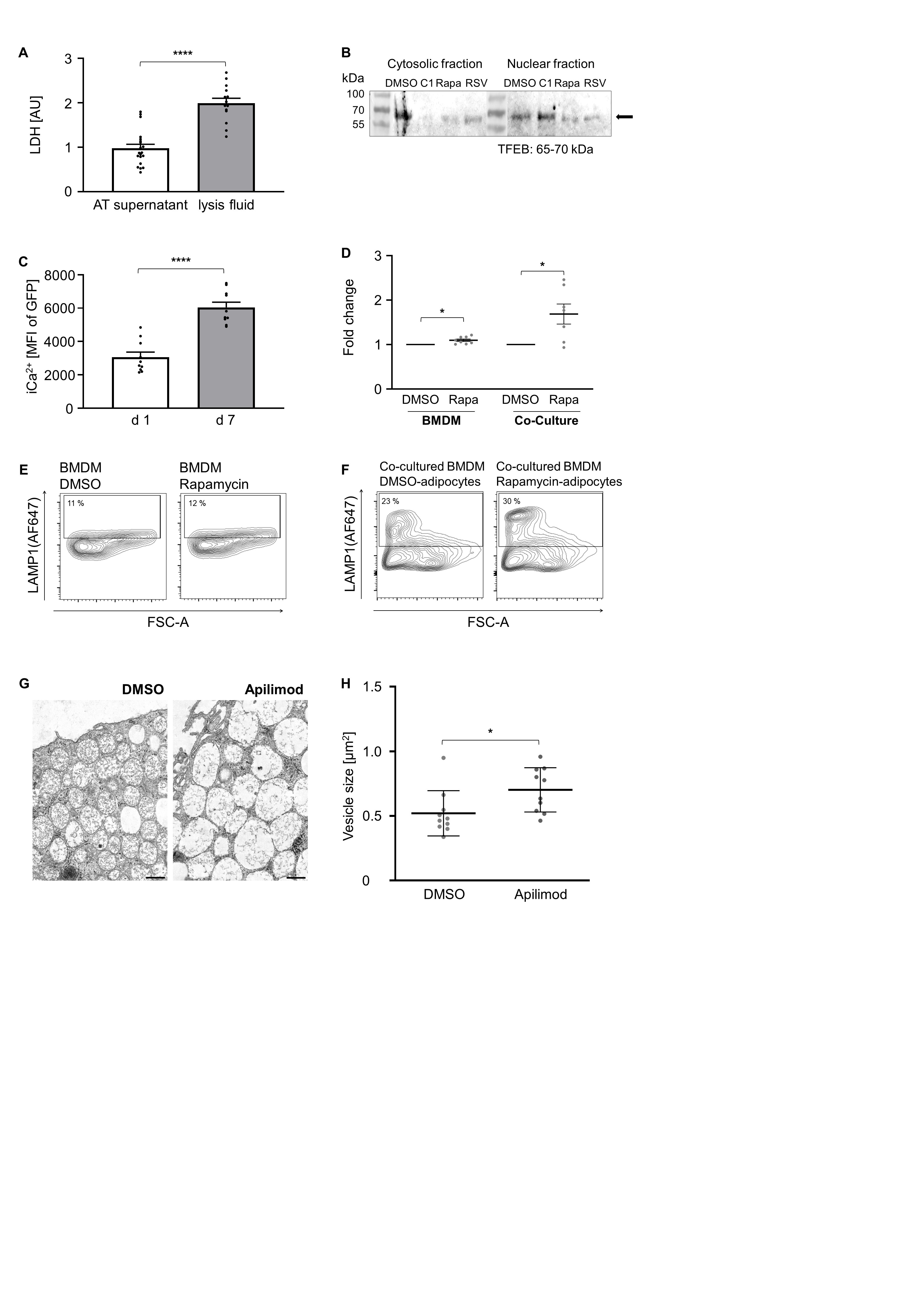

Supplement: Supplementary file 1 — Supplemental Figure 1 [file 41419_2025_8334_MOESM1_ESM.jpg]

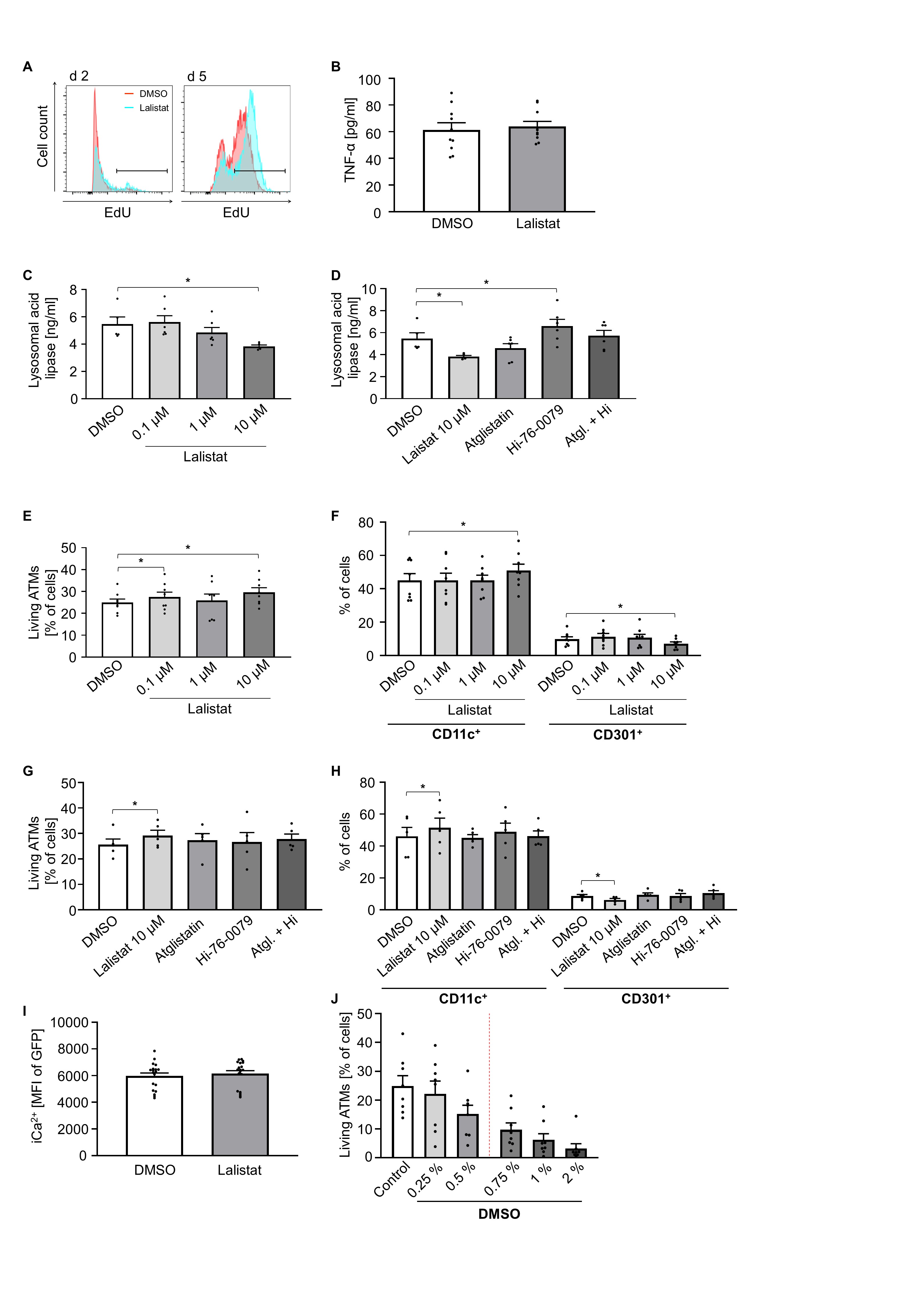

Supplement: Supplementary file 2 — Supplemental Figure 2 [file 41419_2025_8334_MOESM2_ESM.jpg]

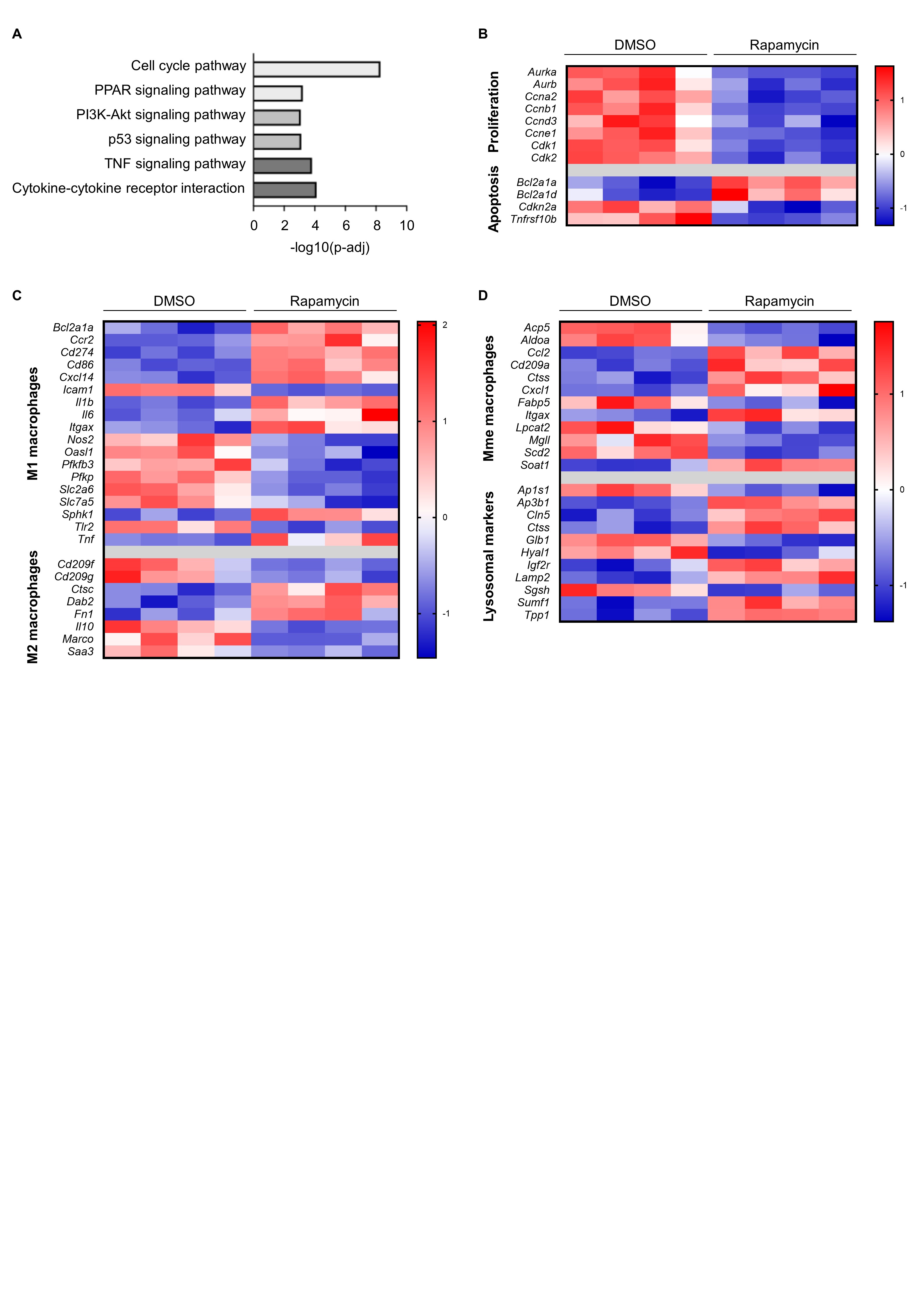

Supplement: Supplementary file 3 — Supplemental Figure 3 [file 41419_2025_8334_MOESM3_ESM.jpg]

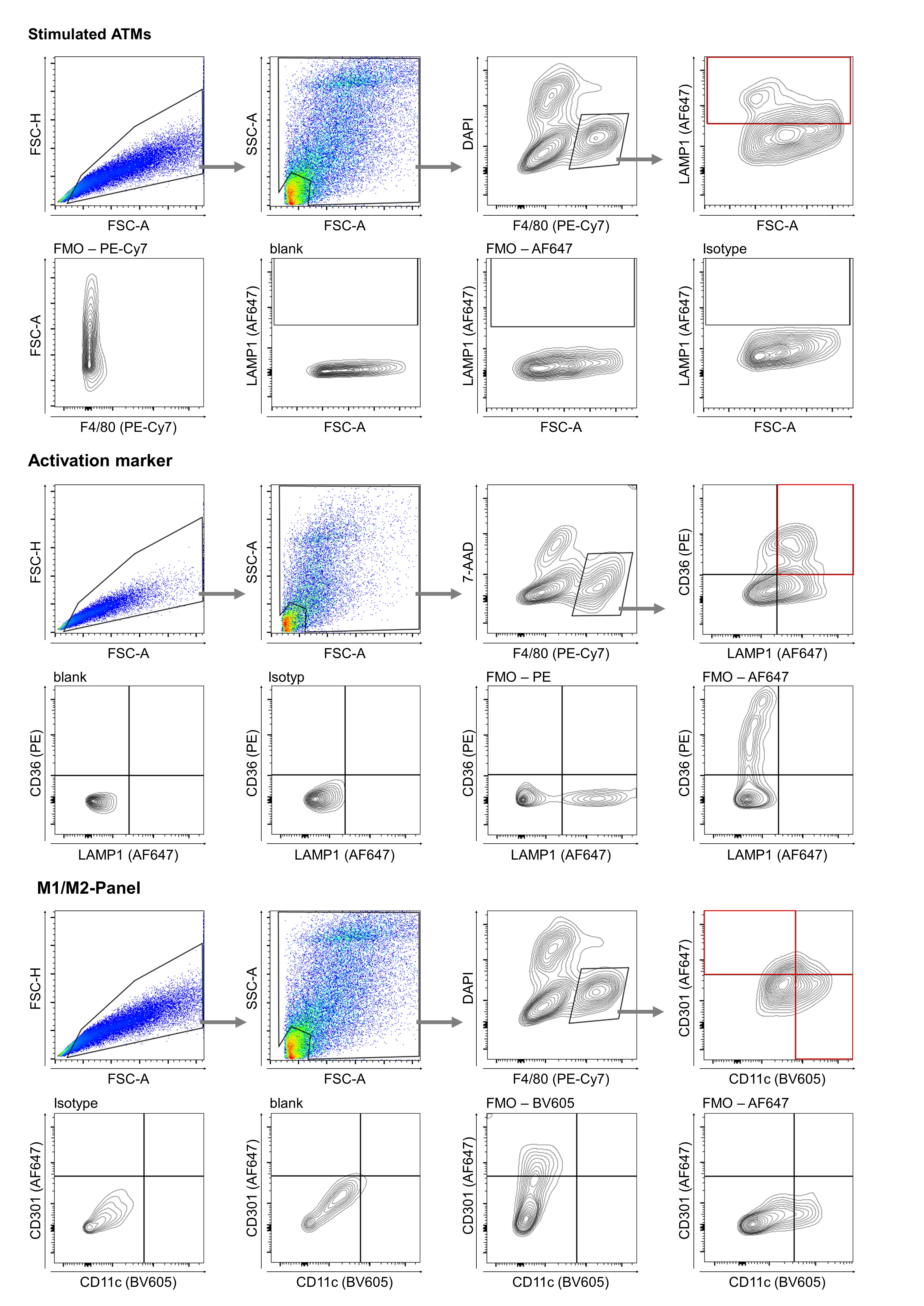

Supplement: Supplementary file 4 — Supplemental Figure 4 [file 41419_2025_8334_MOESM4_ESM.jpg]

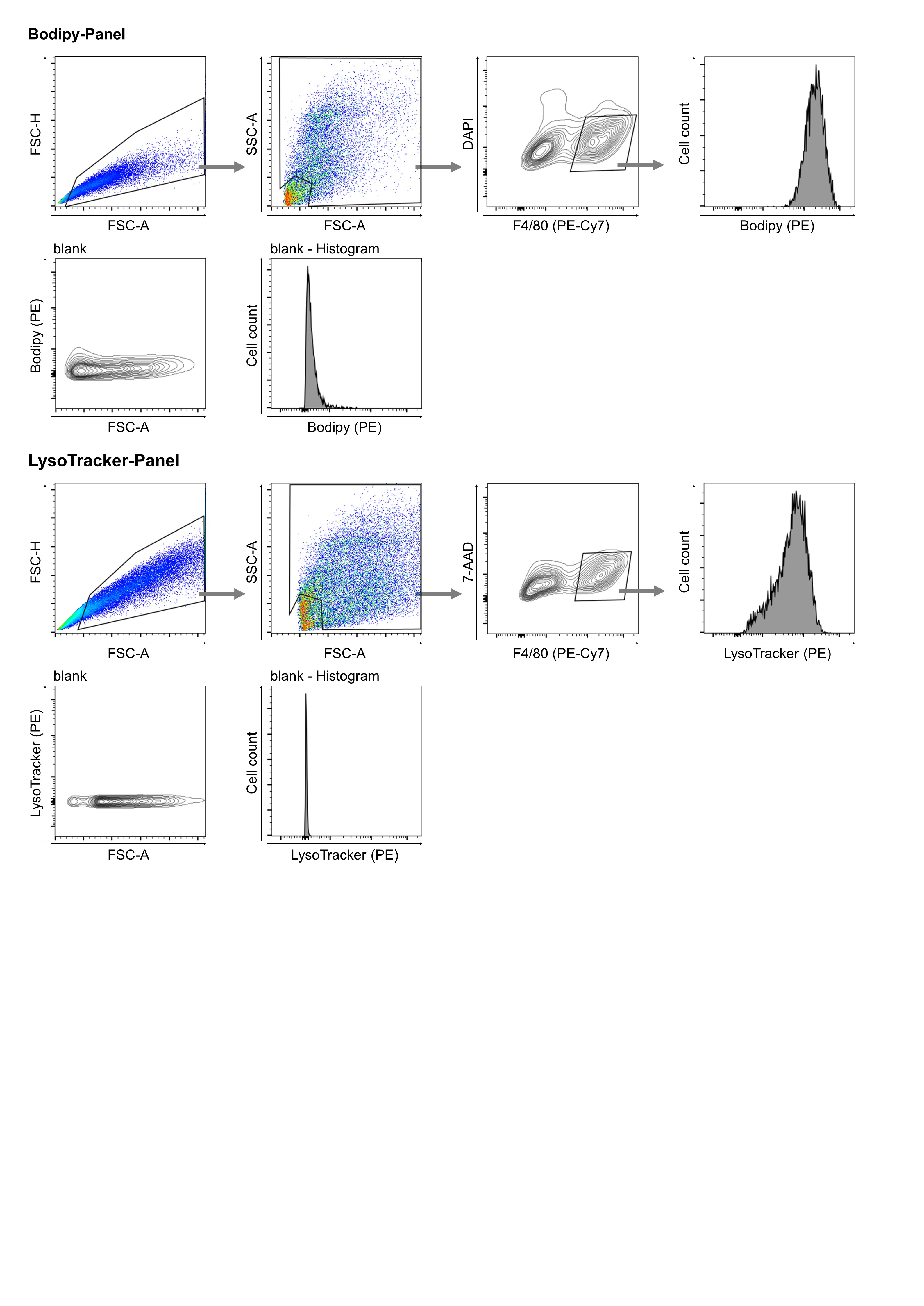

Supplement: Supplementary file 5 — Supplemental Figure 5 [file 41419_2025_8334_MOESM5_ESM.jpg]

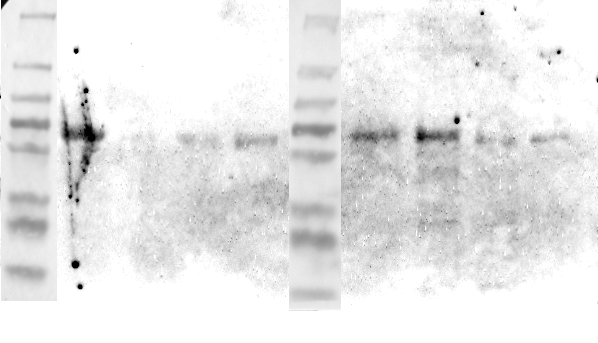

Supplement: Supplementary file 8 — TFEB WesternBlot [file 41419_2025_8334_MOESM8_ESM.png]
