# Supplementary material for: Lysosomal exocytosis by macrophages as a druggable mechanism for anti-inflammatory clearance of dead adipocytes in adipose tissue
Source: Cell Death Dis. 2025 Dec 23;17(1):124. doi: 10.1038/s41419-025-08334-0 (PMC12848297; doi:10.1038/s41419-025-08334-0)
Supplement: Supplementary file 6 — Supplemental Figure Legends [file 41419_2025_8334_MOESM6_ESM.docx]

**Supplemental Figure Legends**

**Supp. Fig. 1. A** Total LDH release: lysed explants as positive control compared with untreated AT explants culture supernatant determined by LDH Assay. **B** Western Blot of cytosolic and nuclear fraction of SVF with anti-TFEB-antibody (65-70 kDa) of DMSO, C1, Rapamycin (Rapa) and Resveratrol (RSV) treated samples. **C** Flow cytometry data of intracellular calcium (iCa^2+^) shown as MFI of GFP in untreated ATMs of CalMac mice at day 1 and day 7. (n=10) **D** Flow cytometry data both of LAMP1 positive BMDMs, treated with DMSO or Rapamycin, and LAMP1 positive BMDMs, co-cultivated with previously treated (DMSO and Rapamycin) murine adipocytes. Data shown as fold change of DMSO-control. (n=8; n=7) **E, F** Representative flow cytometry plots, F4/80^+^ / DAPI^-^ cells further analyzed as LAMP1 positive BMDMs, (**F**) BMDMs were co-cultivated with previously treated (DMSO and Rapamycin) murine adipocytes. **G** Representative electron-microscopy images of DMSO and Apilimod treated ATMs. **H** Vesicle size of lysosomes in DMSO and Apilimod treated ATMs. (n=10) All data shown as mean ± SEM, n indicates the number of mice, p-value < 0.05 statistically significant (*p < 0.05; **p < 0.01; ***p < 0.001; ****p < 0.0001)

**Supp. Fig. 2. A** Representative histogram of EdU proliferation assay of DMSO (red) and Lalistat-2 (blue) samples at day 2 and day 5. **B** TNF-α in Lalistat-2 treated AT supernatant was determined by ELISA. (n=5) **C** Lysosomal acid lipase in AT supernatant of Lalistat-2 concentration series determined by ELISA **D** Lysosomal acid lipase in AT supernatant of Lalistat-2, Atglistatin, Hi-76-0079 and DMSO treated AT were determined by ELISA. (n=6) **E, F** Flow cytometry data of Lalistat‑2 concentration series, F4/80^+^ / DAPI^-^ cells (=living ATMs) further analyzed as CD11c^+^or CD301^+^ ATMs. (n=8) **G, H** Flow cytometry data of Lalistat-2, Atglistatin, Hi-76-0079 and DMSO treated AT, F4/80^+^ / DAPI^-^ cells further analyzed as CD11c^+^ or CD301^+^ cells. (n=5) **I** Flow cytometry data of intracellular calcium, shown as MFI of GFP in ATMs of CalMac mice of DMSO and Lalistat-2 samples. (n=19) **J** Flow cytometry data of living ATMs of DMSO concentration-series. (n=8) All data shown as mean ± SEM, n indicates the number of mice, p-value < 0.05 statistically significant (*p < 0.05)

**Supp. Fig. 3 A Kegg Pathway Analysis** Shown are representative significant regulated pathways (p-adj. < 0.01) of KEGG pathway analysis of significant DEGs of Rapamycin samples. (n=4) **B, C, D** Heatmaps, with z-scores of significant DEGs in Rapamycin samples compared to DMSO control (p-adj. < 0.01 and log2(FC) > 0.5 or < -0.5). (n=4)

**Supp. Fig. 4 and 5.** Gating strategy of flow cytometry samples and Isotypes, blanks and Fluorescence-minus-one (FMO) of flow cytometry samples
