# Supplementary material for: Lysosomal exocytosis by macrophages as a druggable mechanism for anti-inflammatory clearance of dead adipocytes in adipose tissue
Source: Cell Death Dis. 2025 Dec 23;17(1):124. doi: 10.1038/s41419-025-08334-0 (PMC12848297; doi:10.1038/s41419-025-08334-0)
Supplement: Supplementary file 7 — Table 1 Supplemental information [file 41419_2025_8334_MOESM7_ESM.docx]

**Table 1:** Supplemental Information

| **Antibody/Dye** | **Concentration** | **Company** | **Item number** |
| --- | --- | --- | --- |
| CD107a (LAMP1) – AF647 | Staining: 1:100  Flow cytometry: 1:200 | Biolegend | 121610 |
| CD107b (LAMP2) – AF647 | Staining: 1:100 | Biolegend | 108512 |
| F4/80 – PE-Cy7 | Flow cytometry: 1:200 | Invitrogen | 25-4801-82 |
| CD206 – Pacific Blue | Flow cytometry: 1:50 | Biorad | MCA2235PB |
| CD11c – BV605 | Flow cytometry: 1:100 | Biolegend | 117334 |
| CD36 – PE | Flow cytometry: 1:400 | Biolegend | 102606 |
| CD301 – AF647 | Flow cytometry: 1:50 | Biorad | MCA2392A647 |
| F4/80 – AF647 | Flow cytometry: 1:100 | Invitrogen | MF48021 |
| CD16/32 | Flow cytometry: 1:100 | Invitrogen | 14-0161-82 |
| Bodipy 558/568 C12 | Flow cytometry: 1:10000  Staining: 1:2000 | Invitrogen | D3835 |
| LysoTracker – AF647 | Flow cytometry: 1:20000  Staining: 1:10000 | Invitrogen | H34477 |
| DAPI | Flow cytometry: 0.2 µg/ml | Thermo Fisher | 62248 |
| 7-AAD | Flow cytometry: 1:100 | BD Pharmingen | 559925 |
| TFEB Antibody | Western Blot: 1:5000 | Bethyl Laboratories | A303-673A |
| Lammin B1 (host rabbit) | Western Blot: 1:1000 | abcam | Ab133741 |
| GADPH (host mouse) | Western Blot | Fitzgerald | 10R-G109a |
|  | | | |
| **Chemicals/ Reagents** | **Concentration** | **Company** | **Item number** |
| RPMI-Media 1640 |  | Sigma Aldrich | R8758 |
| Fetal Calf Serum | 1:10 | Gibco | 10270106 |
| Penicillin/Streptomycin (10.000 U/ml) | 1:100 | Gibco | 11548876 |
| PBS (-Mg, -Ca) |  | Gibco | 14190169 |
| HBSS (-Mg,-Ca) |  | Gibco | 14170138 |
| Bovine Serum Albumin | 3 % | Sigma Aldrich | A9647 |
| Collagenase Type 2 | 1 mg/ml, 315 U/mg | Worthington | LS004176 |
| Zinc chloride | 0.8 mM | Sigma Aldrich | Z0152 |
| HEPES | 13 mM | Roth | HN77.2 |
| Zinc formaline |  | Polyscience | 21516-3.75 |
| Triton X-100 | 1 % | Carl Roth | 3051.2 |
| Protease inhibitor mixture (100X) | 1:100 | Cell Signaling Technology | #5871 |
| SuperSignal™ West Pico PLUS Chemiluminescent Substrate |  | Thermo Fisher | 34580 |
| Bio-Rad Protein Assay Dye Reagent Concentrate | 1:5 | Biorad | #5000006 |
| Pierce™ ECL Western Blotting-Substrat |  | Thermo Fisher | 32106 |
|  | | | |
| **Treatment** | **Concentration** | **Company** | **Item number** |
| DMSO | 0.5 % / 0.05 % | Sigma Aldrich | D2438-5X10ML |
| Vacuolin | 5 µM | Cayman Chemicals | 20425 |
| Apilimod | 0.5 µM | Sigma Aldrich | SML2974-5MG |
| Lalistat-2 | 10 µM / (1 µM / 0.1 µM) | Sigma Aldrich | SML2053-5mg |
| ML-SI3 | 10 µM | MedChem Express | HY-134819A-5mg |
| ML-SA1 | 10 µM | Sigma Aldrich | SML0627-25mg |
| C1 | 2 µM | TargetMol | TMO-T8055-2mg |
| Rapamycin | 0.5 µM | TargetMol | TMO-T1537-25mg |
| Resveratrol | 5 µM | TargetMol | TMO-T1558-50mg |
| Atglistatin | 50 µM | MedChem Express | HY-15859 |
| Hi-76-0079 | 25 µM | MedChem Express | HY-153523 |
|  | | | |
| **Assay kits and others** | **Concentration** | **Company** | **Item number** |
| Mouse Lipase A, Lysosomal Acid (LIPA) – 96 tests |  | abbexa | abx573100 |
| Mouse Beta-Hexosaminidase Subunit - 96 tests |  | abbexa | abx254518 |
| CyQUANT LDH Cytotoxicity Assay Kit |  | Invitrogen | C20301 |
| BD OptEIA™ Mouse TNF ELISA Kit |  | BD Bioscence | 560478 |
| Click-iT™ Plus EdU Alexa Fluor 350 Flow Cytometry Assay Kit | EdU: 5 µM | Invitrogen | C10632 |
| 4–15% Mini-PROTEAN® TGX Stain-Free™ Protein Gels, 10 well, 50 µl |  | Biorad | #4568084 |
| TC-Insert for 6-well-plates |  | Sarstedt | 83.3930.040 |
| Millicell Cell Culture Insert, 30 mm, hydrophilic PTFE, 0.4 µm |  | Merck Millipore | PICM0RG50 |
| TPP tissue culture test plate 6 |  | TPP | 92006 |
|  | | | |
| **Mice** | | | |
| **Gender** | **Average Age [weeks]** | **Mean Body weight [g]** | **Mean eWAT weight [g]** |
| male | 29 | 35.2 | 1.07 |
